# The Influence of a Polymer Powder on the Properties of a Cold-Recycled Mixture with Foamed Bitumen

**DOI:** 10.3390/ma12244244

**Published:** 2019-12-17

**Authors:** Przemysław Buczyński, Marek Iwański

**Affiliations:** Department of Transportation Engineering, Faculty of Civil Engineering and Architecture, Kielce University of Technology, Al. Tysiąclecia Państwa Polskiego 7, 25-314 Kielce, Poland; iwanski@tu.kielce.pl

**Keywords:** polymer, redispersible polymer powder, foamed bitumen, recycling, cold mixtures, cold recycled mixture

## Abstract

The paper investigates the influence of redispersible polymer powder (RPP) on the physical and mechanical properties of a cold-recycled mixture with foamed bitumen (CRM-FB). Four types of RPP with a varied chemical base were used: VA-VeoVA, VA-VeoVa-Ac, EVA and VA/VV/E/Ac. The polymer powder-modified cold recycled mixture with foamed bitumen, (P)CRM-FB, was composed of 45.8% reclaimed asphalt pavement (RAP), 45.8% natural aggregate (VA), 3.0% Portland cement CEM I 42,5R, 3.0% foamed bitumen 50/70 and 3.0% RPP, all dosed by weight. The reference mixture, (R)CRM-FB, served as a reference point for comparison. It was found that RPP improved the workability of the CRM-FB mixture. This results in a reduced number of compaction cycles and lower energy needed to obtain the air void content as in the reference mixture. In addition, the RPP modifier markedly increased the CRM-FB mixture cohesion (*ITS_DRY_*) and strength, by approximately 40–70%, depending on the RPP used. These findings are particularly important for CRM-FB mixtures designed for road bases. The present investigations confirmed the improvement of the CRM-FB mixture parameters after the modification with RPP, regardless of the powder type used.

## 1. Introduction

Cold deep in place recycling is an established road rehabilitation solution used in Poland [1,2] and worldwide [3,4]. The durability and fatigue life of cold, half-warm [5] and hot mixture asphalt pavements depend on multiple factors. The skid resistance [6] is responsible for the safety of users, and the fatigue life of pavement layers depends on the type of binders [7,8,9], additives [10] and mineral components used, that is, aggregate [11] and reclaimed asphalt pavement (RAP) [12]. Common additives in cold deep recycled mixtures with foamed bitumen and bitumen emulsion include: cement, lime, ash (waste from cement production) [13] and hydraulic binders, which generally increase cohesion, hence, durability, and in some cases improve the resistance to weather (moisture, interaction of moisture and frost). The composition of a cold-recycled mixture may cause many problems. As demonstrated by the authors of [14], incorrectly designed cold-recycled mixtures may become excessively stiff, which leads to the formation of shrinkage cracks in the base course. In turn, an insufficient amount of cement may reduce indirect tensile strength and thus shorten the fatigue life [15]. Therefore, finding a solution to improve fatigue life without increasing the stiffness modulus of the cold-recycled mixture with foamed bitumen and cement seems critical.

Polymers impart resilience to construction materials and improve their ductility by significantly altering their properties. Mazurek and Iwański reported the benefits of using highly modified bitumen in stone mastic asphalt (SMA) mixtures [16]. Improved properties of polymer-modified bitumen have been confirmed in a number of other studies [17,18,19]. Polymers find many applications in cement concrete and cement-based materials. Various forms and types of polymers are used both to modify cement concrete mixtures and to produce polymer-cement concretes (PCC), polymer-impregnated concretes (PIC) and polymer concretes (PC) [20]. In [21], Kim et al. presented an application of EVA polymer to the production of polymer-modified cement mortar (PCM), a widely used repair material for reinforced concrete (RC) structures due to its excellent strength and durability. Successful testing of the addition of polymers to cement concrete was described by Gutarowska et al. [22] and Shen et al. [23].

Considering the results of the studies above, supporting the benefits from adding polymers to cement concrete, paving-grade bitumen and asphalt mixtures, it seems necessary to determine the effects of polymers on the properties of cold-recycled mixtures with foamed bitumen (CRM-FB).

The main aims of the article are to explain how the CRM properties are modified by the addition of redispersible polymer powders (RPPs) and to confirm that the transfer of polymer properties to the CRM properties in terms of increased cohesion, increased elasticity of the mixture and increased ductility will reduce the risk of shrinkage cracks in road bases containing hydraulic binder. The results of these analyses will contribute to broadening both the scope of knowledge on the cold deep recycling with foamed bitumen and the scope of their application to road rehabilitation and reconstruction.

## 2. Aim and Scope of Study

**Figure 1:**
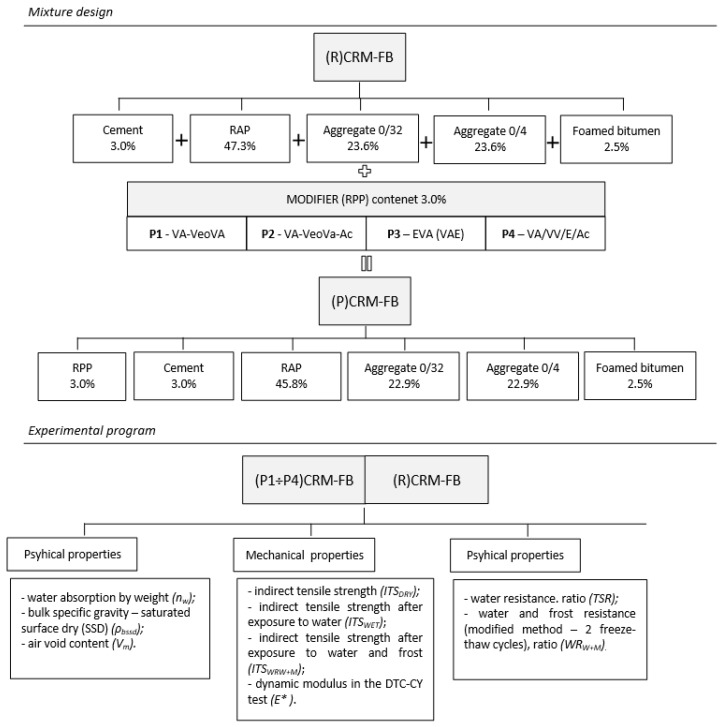
Experimental design.

The present article aimed to investigate the changes in physical and mechanical properties of a cold recycled mixture with foamed bitumen (CRM-FB) after the modification of its structure with a redistributable polymer powder (RPP). The investigations of mechanical properties were focused on the assessment of indirect tensile strength (ITS) and properties related to the stiffness of the recycled mixture. Considering the mechanism of polymer interaction with the properties of building materials, the application of RPPs in the composition of a CRM-FB mixture should increase the cohesion of the CRM-FB without increasing its stiffness. Confirmation of this relationship will show that the modification of the CRM-FB structure has been achieved. Statistical tools in the form of multiple comparison tests with the Tukey test were used. The grouping evaluation showed similar effects of polymer powders with different chemical bases on a given characteristic. The results revealed similarities and differences between the modifiers in terms of the effect on the properties of CRM-FB mixtures. Figure 1 shows a block diagram of the experimental design.

## 3. Materials

### 3.1. Redispersible Polymer Powder

Four types of redispersible polymer powder with different base polymers were used in the tests. They included thermoplastic copolymers (plastomers), where the protective colloid was polyvinyl alcohol (PVA) for preventing the particles from coalescing during the spray drying of the dispersion [20]. Table 1 compiles the most important information about RPPs, adapted from [24]. 

**Figure 2:**
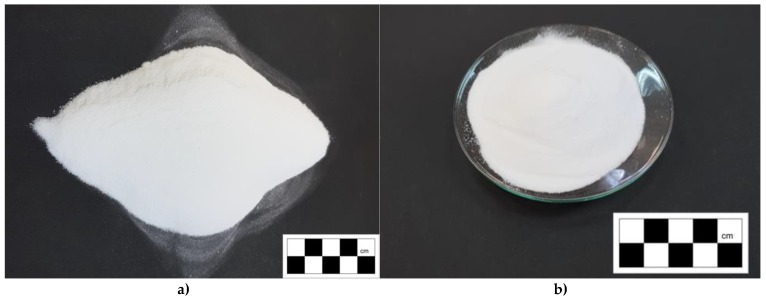
Redispersible polymer powders: (**a**) ethylene-vinyl acetate copolymer (EVA) and (**b**) vinyl acetate-vinyl versatate copolymer (VA-VeoVA).

**Figure 3:**
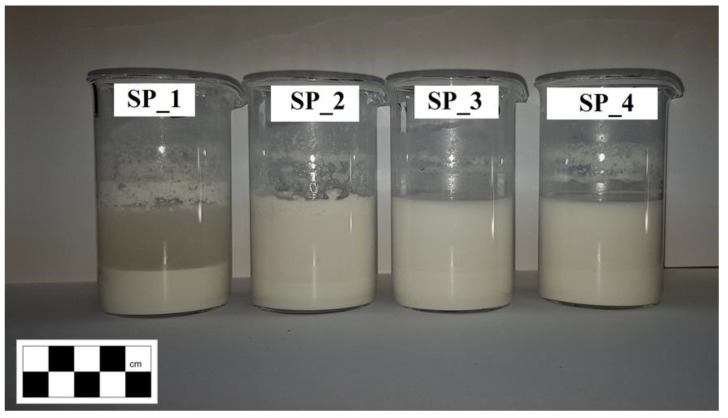
Redispersible polymer powders—polymer emulsion.

**Figure 4:**
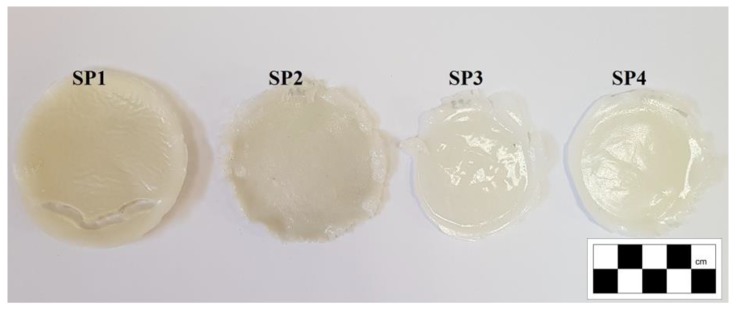
Continuous polymer phase.

The polymers used in the experiment had the form of a redispersible powder obtained through the evaporation of water from polymer emulsions. Redispersible polymer powders are polymer emulsions transformed into a powder through a series of processes such as heat treatment, pressure and spray drying [26]. When mixed with water, the powder re-disperses in water back into polymer emulsion. Examples of redispersible powders under analysis are shown in Figure 2 (powder), Figure 3 (after mixing with water at 24 h of sedimentation) and Figure 4 (continuous polymer phase).

Figure 3 shows the de-emulsification and (in the case of SP2) flocculation of the RPPs 24 h after mixing with water. It should also be noted that in each case, the liquid phase and the solid phase were separated as a result of sedimentation (Figure 3). Similar phenomena were observed by the authors of [27]. The heaviest particles, in the form of the base polymer, sink to the bottom, whereas the protective colloid, in the form of polyvinyl alcohol (PVA), is dissolved in water [28], coloring it white.

A uniform continuous phase of the base polymer was achieved after the sedimented material was dried (Figure 4). A trace amount of the protective colloid was found on the surface in the form of a white powder.

The protective colloid content was also determined during the tests as the parts soluble in water. This was done by determining the weight loss after the polymer dissolved in the water and dried to constant weight in a dryer with a fan at 105 ± 5 °C. The results of the analysis are given in Table 2. 

The contents of the protective colloid/parts soluble in water (Table 2) were very similar, regardless of the RPP type analyzed.

**Figure 5:**
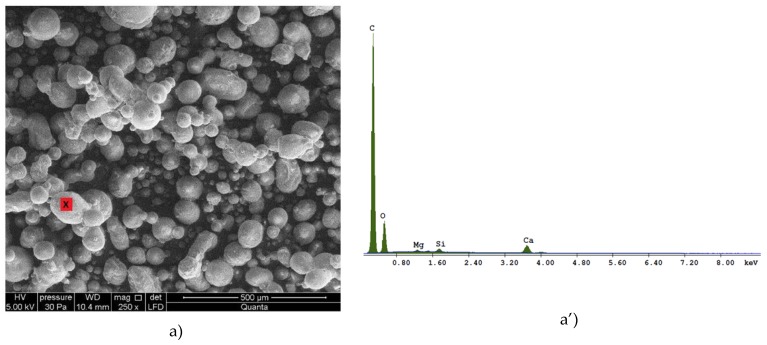

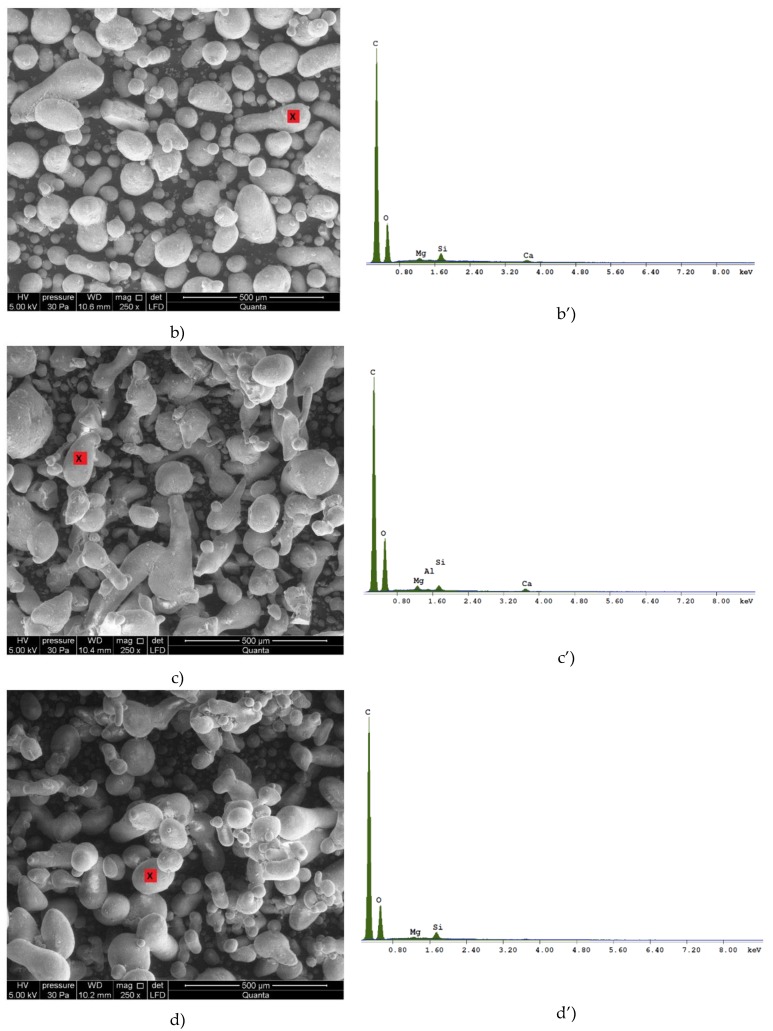
Microstructure of RPPs: (**a**) SEM for P1 (VA-VeoVA); (**a’**) EDX graph for P1 (VA-VeoVA) (**b**) SEM for P2 (VA-VeoVA-A); (**b’**) EDX graph for P2 (VA-VeoVA-A); (**c**) SEM for EVA (VAE); (**c’**) EDX graph for EVA (VAE); (**d**) SEM for VA/VV/E/Ac; (**d’**) EDX graph for VA/VV/E/Ac.

Scanning electron microscopy/energy dispersive X-ray spectrometry (SEM/EDS) was used for in-depth assessment of the microstructure and chemical composition of the RPPs. The material was examined under low-vacuum conditions (pressure of 30 Pa). Sample images of the RPP microstructure are shown in Figure 5. The point that was analyzed for chemical composition was marked red. Table 3 summarizes the results of chemical analysis.

The morphology of the RPPs showed no relationship between the type of the base polymer and the shape of the powder particles. The shape and size of the powder particles depend on the production process [29,30]. According to the literature [20], the polymer particle size in redispersible powders is usually within the range of 1–10 μm. The shape of the particles was most similar to a sphere with a tendency for elongation. Analysis of the composition showed the predominance of carbon with traces of magnesium, aluminum and silicon.

### 3.2. Aggregate

**Figure 6:**
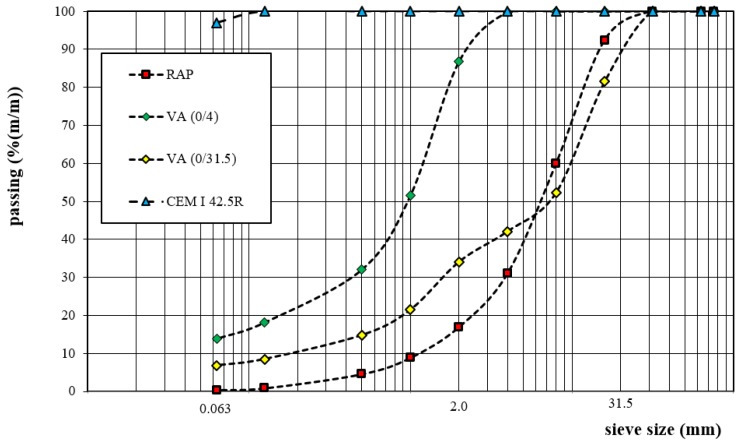
Grading curves of mineral materials.

Two types of mineral component, i.e., natural aggregate and reclaimed aggregate, were used in the CRM-FB mixture. The aggregate was natural crushed fine dolomite 0/4 mm (VA (0/4)) and 0/31.5 mm (VA (0/31.5)). The bulk density of the grains was as follows: 0/4 mm: ρ_a_ = 2.68 Mg/m^3^, 0/31.5 mm: ρ_a_ = 2.80 Mg/m^3^ and reclaimed asphalt pavement (RAP) ρ_a_ = 2.43 Mg/m^3^. The type of aggregate was selected in terms of grain size for the required continuous grading of the asphalt mixture. The grading curves of the mineral components are shown in Figure 6.

Detailed identification of the RAP components was performed focusing on the bitumen type. The tests were carried out in accordance with a series of standards: EN 12697-1 for the amount of soluble asphalt and EN 933-1 + EN 12697-2 for the particle size distribution of the mineral mixture. The analysis of the test results showed 4.8% binder content, 10.9% filler fraction (≤0.063 mm), 35.8% sand (0.063 ÷ 2.0 mm) and 53.3% mastic (≥2 mm). To evaluate the type of bitumen, an appropriate amount of the binder was extracted from the mixture and recovered in a rotary evaporator in accordance with the procedure set forth in EN 12697-3 [31]. Test results of the bitumen samples are compiled in Table 4. 

The results indicate that the RAP contained 35/50 paving-grade bitumen.

### 3.3. Foamed Bitumen

Foamed bitumen was produced with a sol–gel bitumen 50/70. Before foaming, basic parameters of the RAP-extracted bitumen were determined to the relevant standard [32]. Table 5 shows the results.

The use of paving-grade bitumen for foaming, and hence, as a component in CRM-FB mixtures requires that the bitumen foaming characteristics and optimum foaming water content (OFWC) be determined. The amount of water required for bitumen foaming was determined in accordance with the procedure described in relevant guidelines [33]. Additionally, the suitability of the bitumen for foaming is evaluated with respect to two parameters, the maximum expansion ratio (ERm) and half-life H-L [33]. The minimum value of these parameters depends on the temperature of the aggregate surrounded by foamed bitumen [33,34], and it should be equal to:

- ERm ≥ 10 and HL ≥ 8 s for aggregate temperatures from 10 to 15 °C,
- ERm ≥ 8 and HL ≥ 6 s for aggregate temperatures above 15 °C.

**Figure 7:**
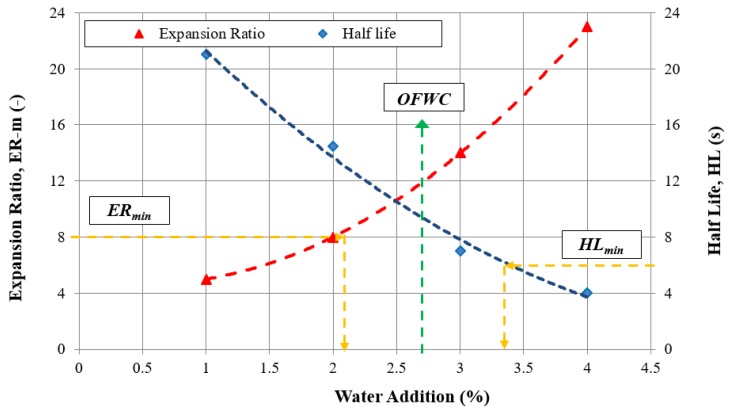
Foaming parameters of paving-grade bitumen 50/70.

Since the mixture was prepared under laboratory conditions, the minimum values were as those for aggregate at ≥15 °C. The optimum foaming water content is indicated in Figure 7.

The optimum foaming water content (OFWC) for the 50/70 paving-grade bitumen determined in accordance with the data in Figure 7 was 2.7%. As indicated by the authors in [35], measurement accuracy is very important for the assessment of OFWC, which is why it is necessary to use state-of-the-art measuring systems [35] or conduct the assessment in such a way as to exclude the effect of external factors.

### 3.4. Hydraulic Binder

The CRM-FB mixture includes Class-I Portland cement with a 42.5 compressive strength and a high early strength (“R”) conforming to EN 197-1 [36]. The basic properties of CEM I 42,5R Portland cement are compiled in Table 6.

## 4. Mixture Design and Sample Preparation

### 4.1. Mixture Design

**Figure 8:**
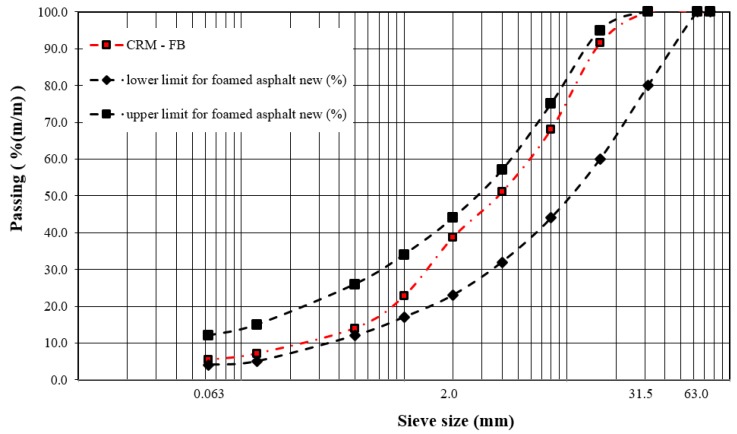
Cold recycled mixture with foamed bitumen (CRM-FB) mixture gradation curve.

For the asphalt mixture, the proportions of the mineral components, 0/4 mm natural dolomite aggregate (VA # 0/4), 0/31.5 mm natural dolomite aggregate (VA # 0/31.5) and 0/31.5 mm RAP (RAP # 0/31.5), were established to ensure conformity with the optimum mixture gradation criterion [33]. In order to achieve the required particle size for the Marshall test [37], the mineral material was passed through a 22.4 mm sieve. The percentage amount of the mineral components with the optimum gradation is given in Table 7 and shown in Figure 8.

The CRM-FB mixture contained 3.0% Class-I 42,5R Portland cement, 2.5% bitumen foam made from 50/70 paving-grade bitumen and 3.0% redispersible polymer powders dosed by weight. The ultimate composition of the mixture is shown in Table 8.

As a result, five cold-recycled mixtures with foamed bitumen were produced. Four of these mixtures—(P1–P4) CRM-FB—contained redispersible polymer powders. The fifth mixture was the RPP-free reference mixture (R)CRM-FB. This approach made is possible to compare and determine the impact of the RPPs on the physical and mechanical properties of CRM-FB mixtures.

### 4.2. CRM-FB Preparation and Curing

The test specimens were prepared in a laboratory mixer with a batch size of 30 kg. Bitumen foam was produced in a laboratory foamer. The optimum moisture content (OMC) in the mixture, as assessed in accordance with EN 13286-2 [38] using the Proctor method, was 5.8%.

Compaction methods varied depending on the test type. For determining the physical and mechanical properties (i.e., bulk density, water absorption, air void content and indirect tensile strength), the impact Marshall compactor [39] was used with 60 blows per minute and 75 blows per side.

For dynamic tests, that is, for complex modulus E* determination [40], in the direct tension–compression test on cylindrical specimens (DTC-CY), a gyratory compactor [41] was used. The settings were chosen according to the literature data [42]. The number of gyrations was established individually for each mixture to obtain the density at which air void content in the CRM-FB mixture was *V_m_* = 10.0%. The required CRM-FB mixture air void content for low volume roads should be within the range of 8–18%, and from 8% to 15% for moderate volume roads [43].

The specimens prepared as described above, irrespective of the type of test performed, were kept at +20 ± 5 °C in the molds during the first day. On the following day, the samples were removed from the molds and kept at relative humidity ranging from 40% to 70% for 14 days until the test.

## 5. Experimental Program

The assessment of the physical and mechanical properties and resistance to weather allowed determining the extent to which redispersible polymer powders affect the properties of the CRM-FB mixture.

### 5.1. Physical Properties

#### 5.1.1. Water Absorption by Weight (*n_w_*)

Water absorption [33] is the amount of mass and volume of water that can be absorbed by a sample immersed in water for 24 h at a temperature of +25 ± 5 °C and then dried to a constant mass. The water absorption (*n_w_*) is calculated in % (m/m) with an accuracy of 0.1% according to the Formula:

(1) $$n_{w}=\frac{m_{1}-m}{m}\cdot 100$$

where: *m*_1_ = mass of water-saturated sample [2] and *m* = mass of a dry sample (g).

#### 5.1.2. Bulk Density—Saturated-Surface-Dry (SSD)

Bulk density—saturated surface dry (SSD) is the mass per unit volume of the sample, including air-filled voids, at a specified test temperature [44]. The bulk density SSD of the sample (*ρ_bssd_*) must be calculated with an accuracy of 0.001 Mg/m^3^ using the following Formula (2):

(2) $$\rho_{bssd}=\frac{m_{1}}{m_{3}-m_{2}}\times \rho_{w}$$

where: *ρ_bssd_* = bulk density (SSD), expressed in megagrams per cubic meter (Mg/m^3^); *m*_1_ = mass of the dry specimen, expressed in grams (g); *m*_2_ = mass of the water saturated specimen, expressed in grams (g); *m*_3_ = mass of the saturated surface dry specimen, expressed in grams (g) and *ρ_w_* = density of water at the test temperature, expressed in megagrams per cubic meter (Mg/m^3^).

#### 5.1.3. Air Void Content (*V_m_*)

Air void content *V_m_* [45] is the volume of air voids in the CRM-FB specimen, expressed as percentage of the overall volume of the specimen, in accordance with Formula (3).

(3) $$V_{m}=\frac{\rho_{m}^{}-\rho_{b}}{\rho_{m}}\cdot 100\%$$

where: *ρ_m_* = CRM-FB mixture density (Mg/m^3^) and *ρ_bssd_* = CRM-FB mixture bulk density (Mg/m^3^).

### 5.2. Mechanical Properties

#### 5.2.1. Indirect Tensile Strength (*ITS_DRY_*)

The indirect tensile strength test *ITS_DRY_* [46] was performed on Marshall specimens with a 101.6 ± 0.3 mm diameter and a 62.5 ± 2.5 mm height, cured for 28 days at the relative humidity ranging from 40% to 70% and a temperature of +25 °C. The ITS test is performed by placing the specimen between two plates and subjecting it to a constant load at a rate of advance of 50 ± 2 mm/min. The *ITS_DRY_* is calculated according to Formula (4).

(4) $$ITS_{DRY}=\frac{2\cdot P}{\pi \cdot h\cdot D}$$

where: *P* = maximum force to failure; *h* = height of the specimen and *D* = diameter of the specimen.

#### 5.2.2. Indirect Tensile Strength after Exposure to Water (*ITS_WET_*)

The indirect tensile strength test *ITS_DRY_* [46,47] was performed on Marshall specimens with a 101.6 ± 0.3 mm diameter and a 62.5 ± 2.5 mm height, cured for 28 days at the relative humidity ranging from 40% to 70% and submerged in water at a temperature of +25 ± 5 °C. The test temperature was +25 °C. The *ITS_WET_* is calculated according to Formula (5).

(5) $$ITS_{WET}=\frac{2\cdot P}{\pi \cdot h\cdot D}$$

where: *P* = maximum force to failure; *h* = height of the specimen and *D* = diameter of the specimen

#### 5.2.3. Indirect Tensile Strength after Exposure to Water and Frost (*ITS_WRW+M_*)

The indirect tensile strength test *ITS_WRW+M_* [46,48] was performed on Marshall specimens with a 101.6 ± 0.3 mm diameter and a 62.5 ± 2.5 mm height, cured for 28 days at the relative humidity ranging from 40% to 70% and frozen as per the modified AASHTO T283 test procedure [48]. The modification involved the use of two freeze/thaw cycles [49]. The test temperature was +25 °C. The *ITS_WRW+M_* is calculated from Formula (6).

(6) $$ITS_{WRW+M}=\frac{2\cdot P}{\pi \cdot h\cdot D}$$

where: *P* = maximum force to failure; *h* = height of the specimen and *D* = diameter of the specimen.

#### 5.2.4. Dynamic Modulus in the DTC-CY Test (E*)

In the DTC-CY test [40,50], the specimen is subjected to a cyclic sinusoidal loading that induces low strains from 25 to 50 με. To obtain a correct sine function for the stress–strain relationship in the linear viscoelasticity range, the cylindrical specimen had to be properly prepared. The specimen was glued with epoxy adhesive to steel plates and the sensors were fixed around the specimen with 120° spacing. The tests were performed at −15 °C and 40 °C, and 10 Hz.

### 5.3. Assessment of the Resistance to Weather

#### 5.3.1. Moisture Resistance, Tensile Strength Ratio (*TSR*)

The tensile strength ratio *TSR* [47] is the ratio of the tensile strength of water-conditioned specimen, (*TSR_WET_*), to the tensile strength of unconditioned specimen (*ITS_DRY_*). The *TSR* is calculated according to Formula (7).

(7) $$TSR=\frac{ITS_{WET}}{ITS_{DRY}}$$

#### 5.3.2. Water and Frost Resistance (Modified Method – 2 Freeze/Thaw Cycles), (*WR_W+M_*)

The *WR_W+M_* [47,49] defines a drop in the indirect tensile strength of specimens subjected to water and frost damage (*TSR_WRW+M_*) relative to unconditioned specimens (*ITS_DRY_*). The *WR_W+M_* is calculated according to Formula (8).

(8) $$WR_{W+M}=\frac{ITS_{WRW+M}}{ITS_{DRY}}$$

## 6. Test Results and Analysis

### 6.1. Effect of RPPs on Compactability of CRM-FB Mixtures

**Figure 9:**
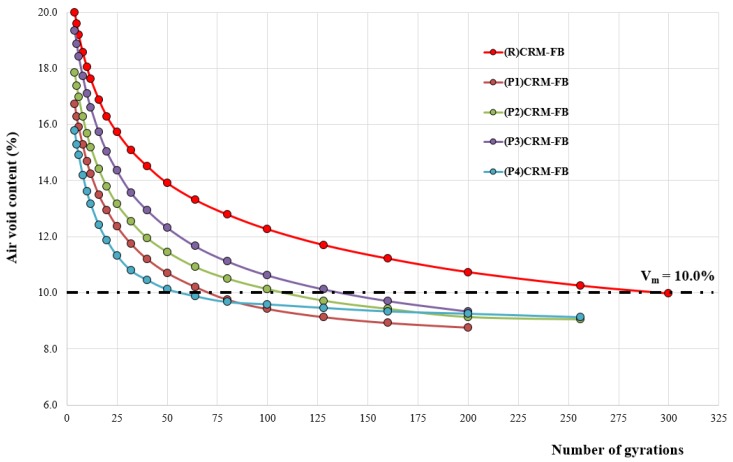
Gyratory compaction of CRM-FB mixture.

The process of gyratory compaction of RPP-modified CRM-FB specimens was different from that for RPP-free CRM-FB specimens in that less energy was needed to obtain the assumed air void content of *V_m_* = 10%. The decrease in air void content as a function of the number of compaction cycles is shown in Figure 9 and Table 9.

It is clear (Figure 8) that RPPs have a positive effect on the compaction process. This can be seen in comparison to the reference mixture ((R) CRM-FB) with 3.0% Portland cement (CEM I 42.5R). Regardless of the type of modifier used, the necessary number of compaction cycles and thus the energy required to achieve the required bulk density (*ρ_bssd_*)/air void content (*V_m_*) was significantly reduced. With modifiers marked as (P2) and (P3), compared to the number of cycles applied to the reference mixture ((R) CRM-FB), three times fewer cycles were necessary to obtain *V_m_* = 10%. For the mixture with modifiers (P1) and (P4), the number of cycles was less than 100. The highest increase in density, and thus a decrease in air void content, was observed in mixtures containing vinyl acetate-vinyl versatate-ethylene-acrylate copolymer (P4). The number of compaction cycles was 58.

In summary, the application of redispersible polymer powders (RPP) was found to have a beneficial effect on the compaction process as it reduced the energy needed to compact the mixture. 

### 6.2. Results of the Tests of Physical Properties, Mechanical Properties and Resistance to Climate Conditions

The mean value for each analyzed parameter was determined with a number of replications, ranging from 4 to 6. The samples of the cold-recycled mixture with foamed bitumen were prepared as described in Section 4.2. The mean values, standard deviation and coefficient of variation for the parameters determined in accordance with the plan of the experiment are given in Table 10 and Table 11.

The test results (Table 10 and Table 11) show high repeatability in relation to individual results. The coefficient of variation was less than 15%, which was satisfactory for a mixture that contains RAP [51]. The stability of the test results regarding the indirect tensile strength (ITS) may be related to the temperature at which the samples were conditioned before the test, i.e., +25 °C. A similar correlation was observed by Gandi et al. [52].

The test results indicate that the majority of the CRM-FB properties analyzed were influenced by the addition of RPP. The variability of this influence was analyzed using a multivariate analysis of variance (MANOVA) [53,54] and Tukey’s multiple-comparison tests.

### 6.3. Multivariate Analysis of Variance and Tukey’s Multiple Comparison Test

For additional evaluation of the effect of modifier type on the physical and mechanical properties and resistance to weather of the CRM-FB mixture, the results were subjected to multivariate analysis of variance (MANOVA) [53,54]. The values of intervals of the tested recycled mixtures were normally distributed. The analysis of variance was conducted for all CRM-FB mixtures. The outcome is shown in Table 12.

The results of the analysis of variance indicate that all of the considered properties were significantly dependent on the type of CRM-FB mixture (modifier type). The *p*-value was smaller than the assumed significance level (α = 0.05), hence with a 5% error the null hypothesis should be rejected [55]. The obtained values differed from each other, and the properties of the CRM-FB mixture are related to the type of modifier used.

To verify the data, a multiple comparison test (Tukey’s test) of the interdependent groups was performed in terms of the modifier type used in the mixture. Significant differences between mean pairs were based on the characteristics obtained from the analysis of variance. This made it easier to identify the differences between the groups and achieve the same significance level for all measurements. The multiple comparison test was preceded by Bartlett’s test for homogeneity of variances in the groups [55].

**Figure 10:**
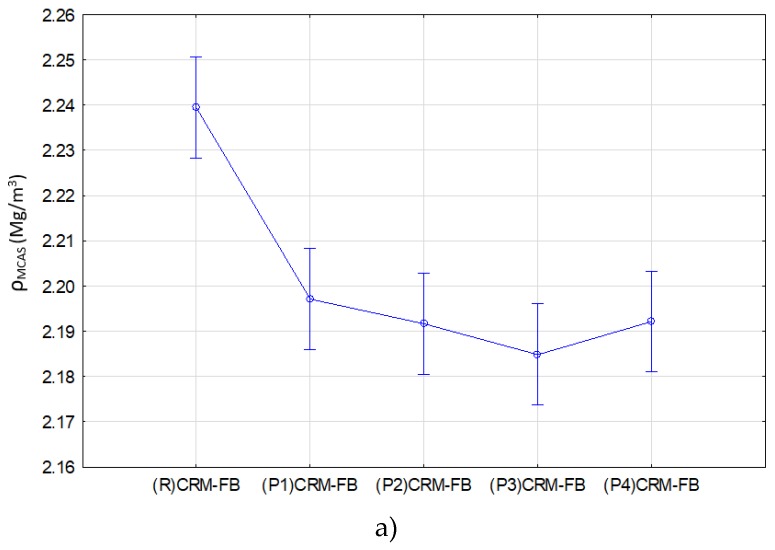

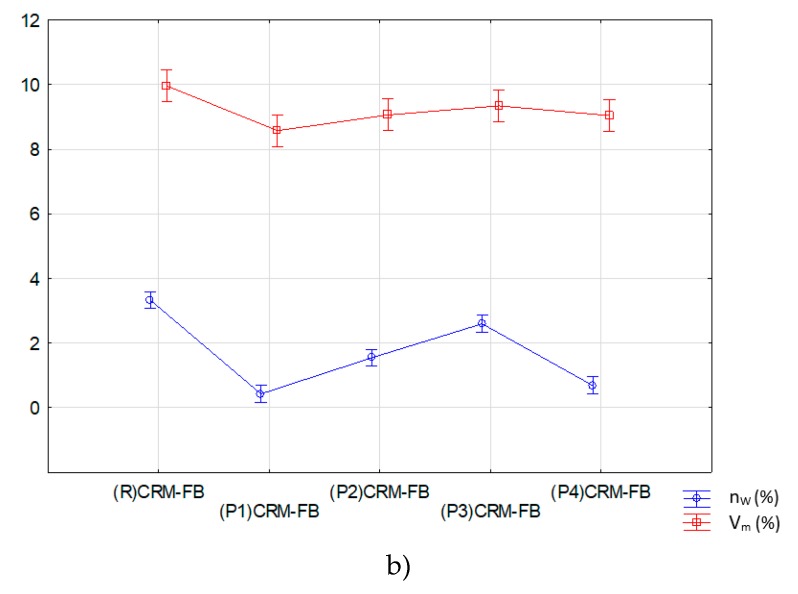
Effect of the modifier type on: (**a**) bulk density (*ρ_MCAS_*) and (**b**) water absorption (*n_w_*), and air void content (*V_m_*).

**Figure 11:**
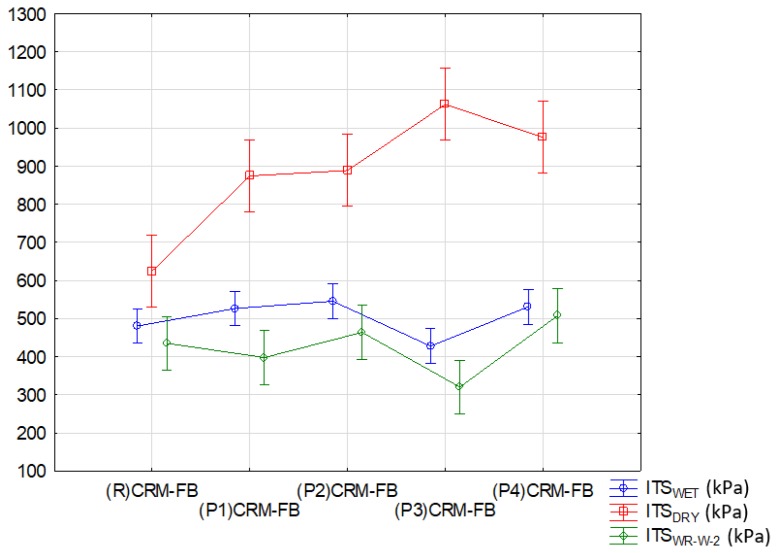
Effect of the modifier type on indirect tensile strength.

**Figure 12:**
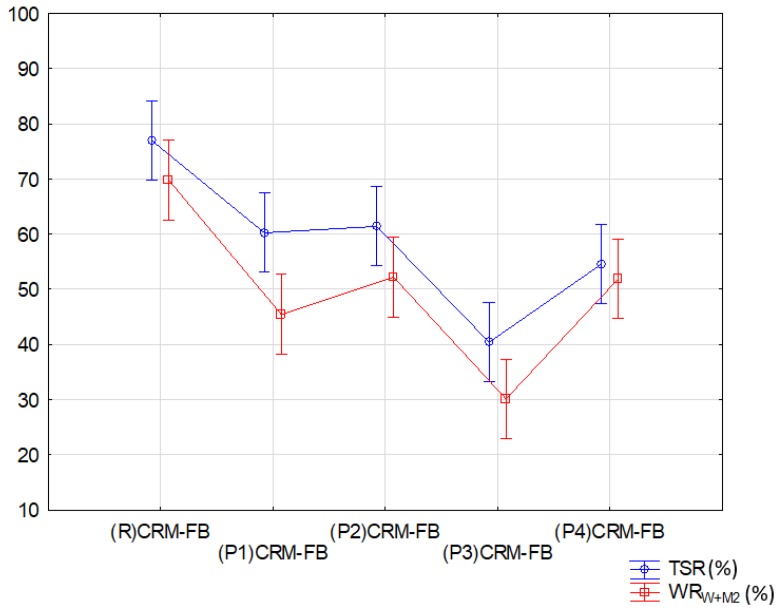
Effect of the modifier type on resistance to weather (*TSR*; *WR_W+M_*2).

The results of multiple comparisons at the 0.05 significance level (Tukey’s test) are summarized in Table 13, Table 14 and Table 15 and shown in Figure 10, Figure 11 and Figure 12. 

Results of the bulk density, water absorption and air void content are shown in Table 13 and Figure 9. The “***” sign in Table 13, Table 14 and Table 15 indicates whether the differences between the means of the parameters determined in the particular group were not significantly different from each other.

Tukey’s test results indicate a significant difference in the effects of the modifier on bulk density (*ρ_MCAS_*), water absorption by weight (*n_w_*) and air void content (*V_m_*). Two similarity groups were identified for the bulk density (*ρ_MCAS_*) and air void content (*V_m_*) characteristics. 

The grouping for bulk density (*ρ_MCAS_*) indicates that the type of modifier did not have a statistically significant effect on bulk density. The mixture that differed from the mixtures with the modifier with respect to bulk density was the reference mixture ((R) CRM-FB).

The results of Tukey’s test regarding water absorption by weight (*n_w_*) indicate that the set of results essentially includes four groups with statistically significant differences. The lowest water absorption demonstrated the CRM-FB mixtures containing the (P1) and (P4) modifiers. As regards the remaining CRM-FB mixtures, the test results suggest that they varied significantly as compared to other mixtures.

Regarding the air void content (*V_m_*), the comparative analyses demonstrated the presence of two groups with statistically significant differences. However, both groups include CRN-FB mixtures with the same modifier type. Mixtures that demonstrate differences but do not belong to the same groups were the ((P1) CRM-FB) mixture with the air void content (*V_m_*) = 8.7%, and the ((R) CRM-FB) mixture with the air void content (*V_m_*) = 10.0%, i.e., the mixtures with the maximum and minimum air void contents.

The results of the analysis for indirect tensile strength (ITS; before and after conditioning) are summarized in Table 14 and illustrated in Figure 11.

Analysis of grouping for indirect tensile strength (ITS) characteristic in terms of conditioning method (Figure 11) indicates that the influence of the modifier was revealed in successive stages of conditioning, that is, exposure to water (*ITS_WET_*) and to the interaction of water and negative temperatures (*ITS_WRW+M_*2). 

As regards the indirect tensile strength (*ITS_DRY_*) of the CRM-FB samples at +25 °C without the impact of weather conditions, two statistically significant groups were identified. The first of these includes all mixtures containing the RPP modifier, and the second group is the reference mixture ((R) CRM-FB). This result of the grouping, with only one mixture in a group, indicates that the mixture is completely different from the remaining mixtures. This is due to the fact that the ITS of the RPP-modified CRM-FB was 40–70% higher (depending on the modifier type) than the ITS for the reference mixture.

The effects of water and the interaction of water and frost resulted in a significant ITS decrease in the CRM-FB specimens, increasing the number of mixtures in the second group. The results of the analysis for *TSR* and *WR_W+M2_* are summarized in Table 15 and illustrated in Figure 12.

The variation results for (*TSR*) and (*WR_W+M_*2) were classified in three groups, irrespective of the type of analyzed indicator/factor describing the impact of weather. Identical behavior with respect to the resistance to moisture damage (*TSR*) and interaction of water and frost (*WR_W+M2_*) was observed in (P1) CRM-FB, (P2) CRM-FB and (P4) CRM-FB mixtures. The third group, similarly to most of the analyzed parameters, included the (R) CRM-FB reference mixture, which was characterized by the greatest variability in comparison with the mixtures containing the modifier.

**Figure 13:**
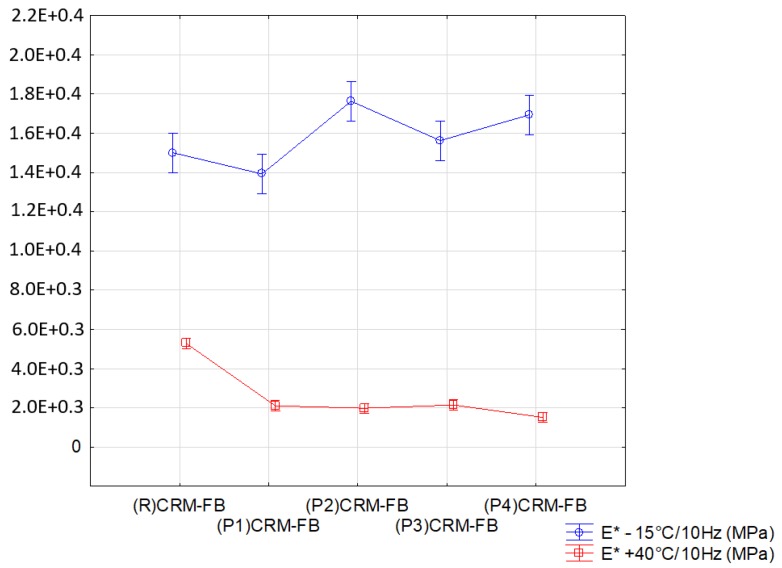
Effect of modifier type on the dynamic modulus.

The results of the analysis for dynamic modulus (E*) are summarized in Table 16 and illustrated in Figure 13.

The grouping obtained in the multiple-comparison tests (Table 12 and Table 15) demonstrated that three groups of similarities existed for the complex modulus (E*) examined at −15 °C and that at +40 °C. The groups established for the analyzed properties were not identical, which indicates that the modifiers had different effects on the mixtures at low and high temperatures. 

### 6.4. Standardization of Test Results

In order to conclusively determine the impact of the RPPs on the properties of CRM-FB mixtures, the test results were standardized. The results obtained on measurement scales of different properties, e.g., complex modulus E* = 16026 MPa, indirect tensile strength *ITS_DRY_* = 800 kPa and air void content *V_m_* = 10%, can be compared if those results are converted into results expressed on a scale with a single, common unit. That is why the test results were standardized. The test results of physical properties, mechanical properties and resistance to weather conditions were converted into a standardized scale using formula (9):

(9) $$Z=\frac{x\;-\;\mu }{\sigma }$$

where: *x* = result achieved on the original measurement scale; *μ* = mean value of the results for a particular property and *σ* = standard deviation of the results for a particular property.

Standardized values of the results for physical and mechanical properties and resistance to weather are either positive or negative, depending on whether the individual values for the particular mixture deviate up or down from the mean level for ta particular property in the group of mixtures. If the values on the standardized scale are equal to zero (“0”), the standardized values are the same as the mean values. Table 17 shows the values after standardization. 

**Figure 14:**
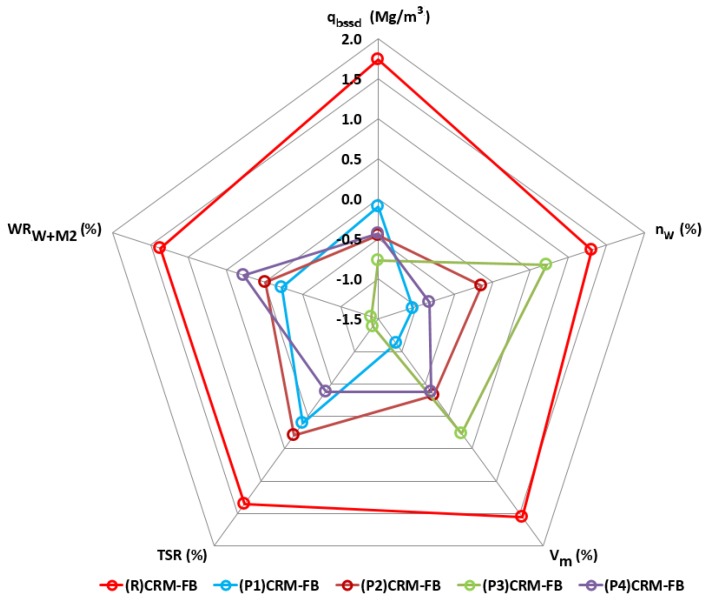
Test results of physical properties and resistance to weather of CRM-FB mixture with RPP (standardized scale).

The effects of the RPPs were evaluated in terms of the physical and mechanical properties of the CRM-FB mixtures. The data obtained (Table 17) were used to present all the standardized results on the radial diagram in Figure 14.

The red color was used in Figure 14 to mark the values and enveloping lines obtained for the reference mixture ((R) CRM-FB). The test results constitute the reference level for the determination of the change in the properties of the CRM-FB against the type of RPP modifier.

Comparison of the (R) CRM-FB mixture values with those for the RPP-modified mixtures (P1–P4) CRM-FB indicates that the addition of the modifier reduces all of the physical parameters and weather resistance. This relationship was observed irrespective of the modifier type. The addition of the modifier increased the water tightness of the mixture, irrespective of the decrease in bulk density. The highest decrease in water absorption (*n_w_*) and air void content (*V_m_*) was observed in the (P1) RCM-FB mixture, where the polymer powder was based on the vinyl acetate-vinyl versatate copolymer. The lowest water tightness was found in the (P3) CRM-FB mixture with an ethylene-vinyl acetate copolymer (EVA). The reduced air void content and water absorption were positive effects of the polymer modifier on the CRM-FB mixture.

A negative impact of the modifier on the properties of the CRM-FB mixture was observed with respect to moisture sensitivity (*TSR*) and resistance to water and frost (*WR_W+M_*2). The modified mixtures showed a greater decrease in indirect tensile strength after conditioning compared with the reference mixture. The (R)CRM-FB mixture showed a 30% decrease in ITS, and had *TSR* = 73% and *WR_W+M_*2 = 70%, whereas in the modified mixtures, the ITS decreased by approximately 50%, with *TSR* = 53% and *WR_W+M_*2 = 44%. The maximum ITS decrease should not exceed 30% (*TSR* and *WR_W+M_*2 ratios should be higher than 70%) [43,47]. The higher decrease in the resistance to the effects of water and to the effect of water and frost in the modified mixtures relative to the reference mixture could be related to the RPP production process. The redispersible polymer powders used in the tests contained a water-soluble protective colloid [27] in the form of polyvinyl alcohol (PVA). After conditioning in water, the colloid might have been washed out of the mixture samples, which increased the air void content and reduced the indirect tensile strength.

**Figure 15:**
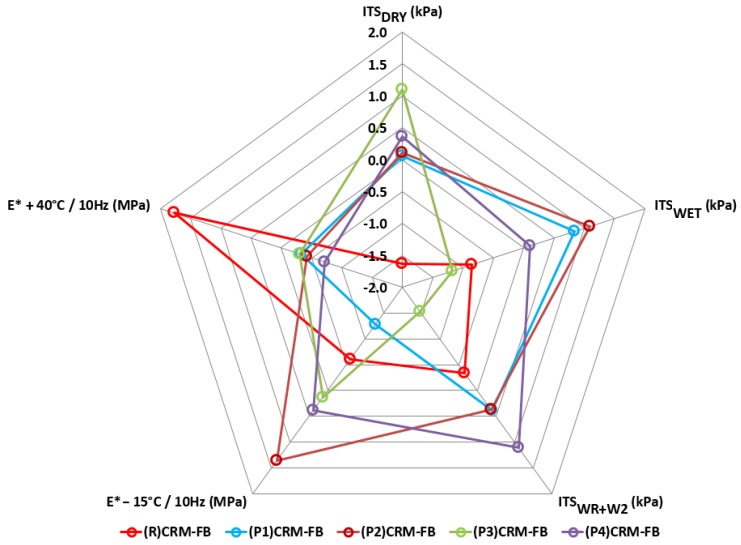
Test results of mechanical properties for the CRM-FB with RPP (standardized scale).

Figure 15 shows the effect of the modifier type on the mechanical properties of the CRM-FB mixture.

The effect of the modifier on the mechanical properties was different from that observed in physical properties and resistance to weather conditions. The modification of the CRM-FB with RPP increased the values of most parameters. The largest increase after the modification was observed with respect to the ITS of samples conditioned at +25 °C. The increase in cohesion (*ITS_DRY_*) relative to the reference mixture was approximately 50%. Higher ITS of CRM-FB mixtures ensures higher structural reliability. This is due to the distribution of stresses in the structure with a base course made of the CRM-FB mixture [56].

According to the literature data [20], the decrease in complex modulus obtained by the cold-recycled mixture at high temperatures represents a correct relationship. No significant differences in the test results were observed for the complex modulus E* at −15 °C, as confirmed by the multiple-comparison analyses (Table 16). At moderate temperatures, complex modulus values decreased [25].

**Figure 16:**
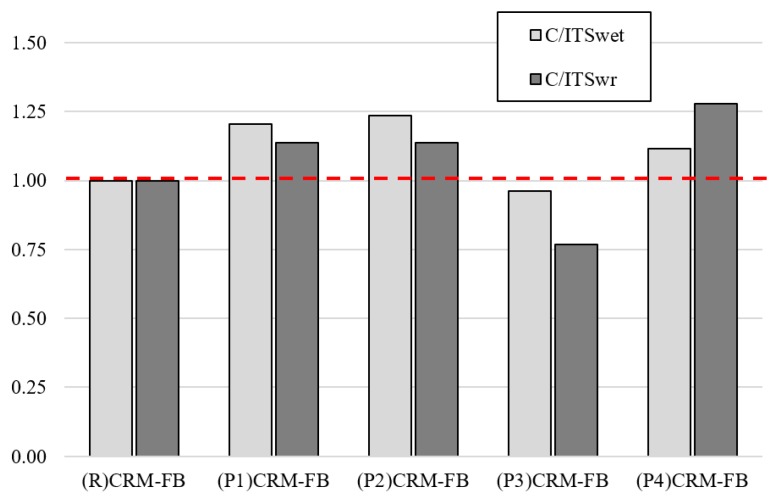
Change ratio of indirect tensile strength after conditioning cycles.

Regarding the unsatisfactory level of resistance to water (*TSR*) and to the interaction of water and frost (*WR_W+M_*2) determined for the modified mixtures, it should be emphasized that the ITS after the conditioning process was much higher than the *ITS_WET_* obtained for the reference mixture. Similar relationships were found for indirect tensile strength after the samples were conditioned by the exposure to water and frost. To illustrate the effect of the modifier on the ITS change against the reference mixture, the C/ITS change ratio was determined in accordance with Formulas (10) and (11):

(10) $$C/ITS_{WET}=\frac{ITS_{WET-SAM.}}{ITS_{WET-REF.}}$$

where: *C/ITS_WET_* = *ITS* change ratio in samples subjected to conditioning through exposure to water; *ITS_WET-REF._* = indirect tensile strength of samples exposed to water (in accordance with the procedure as for the *TSR*) for the reference mixture and *ITS_WET-SAM._* = indirect tensile strength of samples exposed to water (in accordance with the procedure as for the *TSR*) for the RPP-modified mixture;

(11) $$C/ITS_{WR}=\frac{ITS_{WR-SAM.}}{ITS_{WR-REF.}}$$

where: *C/ITS_WR_* = *ITS* change ratio in samples subjected to conditioning through exposure to water and frost (in accordance with the procedure for *WR_W+M_*2); *ITS_WR-REF._* = indirect tensile strength of samples exposed to water (in accordance with the procedure as for the *WR_W+M_*2) for the reference mixture and *ITS_WR-SAM._* = indirect tensile strength of samples exposed to water (in accordance with the procedure as for the *WR_W+M_*2) for the RPP-modified mixture;

The results of the analysis are shown in Figure 16.

The ITS change ratio after the exposure to water or to the interaction of water and frost (Figure 14) indicates that the RPP modifier increased ITS after conditioning relative to a non-modified mixture. That is why the failure to meet the required *TSR* and *WR_W+M_*2 values was not an objective criterion in this case. For the (P1) modifier, i.e., ethylene-vinyl acetate copolymer (EVA), the ITS was lower than in the reference mixture. In the other cases, ITS was 10–28% higher.

## 7. Conclusions

The tests for the effect of the type of RPP modifier on the physical and mechanical properties and the resistance to weather conditions supported the following conclusions:

- The use of all analyzed RPPs had a positive effect on the mechanical properties of CRM-FB mixtures. The modification contributed to the increase in cohesion and flexibility with no stiffening of the base layer, i.e., no increase of the modulus of elasticity was observed.
- Maximum cohesion values described by *ITS_DRY_* parameter, more than 1000 kPa, were observed when EVA (ethylene-vinyl acetate) was used.
- The addition of redispersible polymer powders (RPP) in the CRM-FB reduced the compaction effort needed to obtain optimum bulk density/air void content. The number of compaction cycles was reduced from 300 to about 100.
- The comparison of the physical and mechanical properties and water and frost sensitivity of the CRM-FB mixtures confirmed that the RPP modifier increased the water tightness of the CRM-FB mixture.
- The highest impact of the RPP modifier on the CRM-FB mixture was observed with respect to the indirect tensile strength at +25 °C. The ITS increase ranged from 50% to 70% as compared with the RPP-free reference mixture.
- A negative impact of the modifier was observed regarding the water sensitivity (*TSR*) and the interaction of water and frost (*WR_W+M_*2). The modified mixtures showed a higher ITS reduction after conditioning in comparison with the reference mixture.
- The indirect tensile strength after conditioning ((*ITS_WET_*) and (*ITS_WRW+M_*2)) was higher than that of the reference mixture. It is thus impractical to assess the water sensitivity and the resistance to water and frost of RPP-modified mixtures through *TSR* and *WR_W+M_*2.
- The addition of any RPP under analysis to the CRM-FB resulted in a significant increase in indirect tensile strength (cohesion (*ITS_DRY_*)) accompanied by a simultaneous decrease in complex modulus E*, as compared with the relationships obtained for the reference mixture. This phenomenon is highly desirable in road base courses to provide higher resistance to cracking and mitigate the propagation of existing cracks.

## Figures and Tables

**Table 1:** Redispersible polymer powders used [25].

Code	Description	Name	Bulk Density ISO 679 (g/L)	Base Polymer
P1	VA-VeoVA	vinyl acetate-vinyl versatate copolymer	550–570	vinyl acetate and vinyl versatate
P2	VA-VeoVa-Ac	vinyl acetate-vinyl versatate-acrylate copolymer	530–670	butyl acrylate, vinyl acetate and vinyl versatate
P3	EVA (VAE)	ethylene-vinyl acetate copolymer	450–500	ethylene and vinyl acetate
P4	VA/VV/E/Ac	vinyl acetate-vinyl versatate-ethylene-acrylate copolymer	350–550	ethylene, vinyl acetate, vinyl versatate and butyl acrylate

**Table 2:** Percentage of soluble part in redistributable polymer powder (RPP).

Code	Description	X	s	ν (%)
P1	VA-VeoVA	4.6	0.37	8.0
P2	VA-VeoVa-Ac	4.6	0.42	9.3
P3	EVA (VAE)	5.0	0.30	5.9
P4	VA/VV/E/Ac	4.8	0.34	7.1

**Table 3:** Chemical composition of the RPPs.

Component	Percentage in P1 (%)	Percentage in P2 (%)	Percentage in P3 (%)	Percentage in P4 (%)
C	67.35	67.85	67.67	71.25
O	24.80	27.44	29.13	26.03
Mg	0.53	0.70	0.52	0.40
Si	1.04	2.54	1.65	2.31
Ca	6.28	1.47	0.75	0.0
Al	0.0	0.0	0.29	0.0
∑	100.00	100.00	100.00	100.00

**Table 4:** Test results of the bitumen extracted from reclaimed asphalt pavement (RAP).

Bitumen Parameter	Testing Method	Unit	Result	Stand. Dev.	Coeff. of Variation
Binder content	PN-EN 12697-1	%	4.8	0.1	1.9
Penetration at 25 °C	PN-EN 1426	0.1 mm	35.0	1.3	3.7
Softening temperature	PN-EN 1427	°C	59.8	1.7	2.9
Elastic recovery	PN-EN 13398	%	12.0	1.2	9.6

**Table 5:** Standard test results for the bitumen 50/70.

Parameter	Unit	Standard	Result
			50/70
Penetration	0.1 mm	PN-EN 1426	60.0
Softening point	°C	PN-EN 1427	50.2
Fraass breaking point	°C	PN-EN 12593	−15.0

**Table 6:** Properties of CEM I 42,5R Portland cement.

Property	Testing Method	Unit	Result
Beginning of the setting time	EN 196-3	min	209
Compressive strength	EN 196-1		
at 2 days		MPa	27.2
at 28 days		MPa	55.6
Soundness	EN 196-3	mm	0.8
Specific surface area	EN 196-6	cm^2^/g	3360

**Table 7:** Composition of the mineral mixture.

Mixture	Aggregate Type	(%)	Particle Density ρ_a_ (Mg/m^3^)
CRM-FB	RAP # 0/31.5	50.0	2.43
	VA # 0/4	25.0	2.68
	VA # 0/31.5	25.0	2.80

**Table 8:** Composition of the cold-recycled mixture with polymers—CRM-FB + P.

Component	Percent Content (%)		
	MM	(R) CRM-FB	(P1–4) CRM-FB
Reclaimed asphalt pavement (RAP # 31.5 mm)	50.0	47.3	45.8
Dolomite aggregate (VA # 31.5 mm)	25.0	23.6	22.9
Dolomite aggregate (VA #0/4 mm)	25.0	23.6	22.9
CEM I 42,5R Portland cement	-	3.0	3.0
Redispersible polymer powder (P1–P4)	-	-	3.0
50/70 foamed bitumen	-	2.5	2.5

**Table 9:** Number of compaction cycles for *V_m_* = 10%.

	(R)CRM-FB	(P1)CRM-FB	(P2)CRM-FB	(P3)CRM-FB	(P4)CRM-FB
Number of compaction cycles	300	64	100	128	58

**Table 10:** Test results of physical properties and resistance to weather conditions of the CRM-FB mixture.

Type of Mixture	*q_bssd_* (Mg/m^3^)			*n_w_* (%)			*V_m_* (%)			*TSR* (%)			*WR_W+M_*2 (%)		
	X	s	ν (%)	X	s	ν (%)	X	s	ν (%)	X	s	ν (%)	X	s	ν (%)
(R)CRM-FB	2.239	0.001	0.06	3.34	0.001	0.02	10.0	0.05	0.54	73	6.1	8.3	70	7.7	11.1
(P1)CRM-FB	2.199	0.005	0.20	0.41	0.0004	0.10	8.7	0.26	2.95	59	3.0	5.1	46	1.3	2.9
(P2)CRM-FB	2.192	0.013	0.60	1.54	0.003	0.18	9.1	0.54	6.00	61	4.7	7.7	49	3.7	7.5
(P3)CRM-FB	2.185	0.000	0.00	2.60	0.004	0.15	9.3	0.004	0.04	41	2.0	4.8	29	1.8	6.2
(P4)CRM-FB	2.192	0.003	0.11	0.69	0.0005	0.07	9.0	0.10	1.15	53	4.2	8.0	54	3.9	7.3

Descriptive statistics: X—mean value, s—standard deviation of the sample, *v*—coefficient of variation.

**Table 11:** Results of tests of mechanical properties and resistance to weather conditions of the CRM-FB mixture.

Type of Mixture	*ITS_DRY_* (kPa)			*ITS_WET_* (kPa)			*ITS_WR+W_*2 (kPa)			E* −15 °C/10 Hz (MPa)			E* +40 °C/10 Hz (MPa)		
	X	s	ν (%)	X	s	ν (%)	X	s	ν (%)	X	s	ν (%)	X	s	ν (%)
(R)CRM-FB	642	46	7.10	444	42	9.50	389	58	14.77	14774	462	3.1	5116	342	6.7
(P1)CRM-FB	892	65	7.27	534	11	2.14	443	59	13.33	13626	599	4.4	2033	145	7.1
(P2)CRM-FB	900	14	1.51	548	39	7.21	443	27	6.04	18126	994	5.5	1902	154	8.1
(P3)CRM-FB	1046	68	6.48	426	8	1.93	300	32	10.71	16026	804	5.0	2070	156	7.5
(P4)CRM-FB	937	68	7.24	495	52	10.51	498	47	9.40	16473	934	5.7	1460	115	7.9

Descriptive statistics: X—mean value, s—standard deviation of the sample, *v*—coefficient of variation.

**Table 12:** Analysis of variance (MANOVA).

	*q_bssd_* (Mg/m^3^)	*n_w_* (%)	*V_m_* (%)	*TSR* (%)	*WR_W+M_*2 (%)	*ITS_DRY_* (kPa)	*ITS_WET_* (kPa)	*ITS_WR+W_*2 (kPa)	E* –15 °C/10 Hz (MPa)	E* +40 °C/10 Hz (MPa)
	*p*-Value									
Intercept	<0.001	<0.001	<0.001	<0.001	<0.001	<0.001	<0.001	<0.001	<0.001	<0.001
Modifier type	0.002	0.001	0.028	0.002	0.002	0.003	0.025	0.031	0.006	0.001

**Table 13:** Multiple comparison test of interactions for bulk density, water absorption and air void content.

Subclass Number	Tukey’s HSD Test Homogeneous Groups, α = 0.05								
	Mixture Type	ρ_MCAS_ (Mg/m^3^)		*n_w_* (%)				*V_m_* (%)	
		1	2	1	2	3	4	1	2
4	(P3)CRM-FB	***				***		***	***
3	(P2)CRM-FB	***			***			***	***
5	(P4)CRM-FB	***		***				***	***
2	(P1)CRM-FB	***		***				***	
1	(R)CRM-FB		***				***		***

**Table 14:** Multiple comparison test of interactions for indirect tensile strength.

Subclass Number	Tukey’s HSD Test Homogeneous Groups, α = 0.05						
	Mixture Type	*ITS_DRY_* (kPa)		*ITS_WET_* (kPa)		ITS_WR-W-_2 (kPa)	
		1	2	1	2	1	2
4	(R)CRM-FB		***	***	***	***	***
3	(P1)CRM-FB	***		***	***	***	***
5	(P2)CRM-FB	***		***		***	***
2	(P3)CRM-FB	***			***	***	
1	(P4)CRM-FB	***		***			***

**Table 15:** Multiple comparison test of interactions for weather conditions (*TSR*; *WR_W+M_*2).

Subclass Number	Tukey’s HSD Test Homogeneous Groups, α = 0.05						
	Mixture Type	*TSR* (%)			*WR_W+M_*2 (%)		
		1	2	3	1	2	3
4	(R)CRM-FB			***			***
3	(P1)CRM-FB	***			***	***	
5	(P2)CRM-FB	***			***		
2	(P3)CRM-FB		***			***	
1	(P4)CRM-FB	***	***		***		

**Table 16:** Multiple comparison test of interactions for the dynamic modulus.

Subclass Number	Tukey’s HSD Test Homogeneous Groups, α = 0.05						
	Mixture Type	E* –15 °C/10 Hz (MPa)			E* +40 °C/10 Hz (MPa)		
		1	2	3	1	2	3
4	(R)CRM-FB	***	***				***
3	(P1)CRM-FB	***			***		
5	(P2)CRM-FB			***	***	***	
2	(P3)CRM-FB	***	***	***	***		
1	(P4)CRM-FB		***	***		***	

**Table 17:** Standardized test results for the analyzed properties of CRM-FB mixtures.

Type of Mixture	*Z*									
	*q_bssd_* (Mg/m^3^)	*n_w_* (%)	*V_m_* (%)	*TSR* (%)	*WR_W+M_*2 (%)	*ITS_DRY_* (kPa)	*ITS_WET_* (kPa)	*ITS_WR+W_*2 (kPa)	E* –15 °C/10 Hz (MPa)	E* +40 °C/10 Hz (MPa)
(R)CRM-FB	1.7	1.3	1.6	1.4	1.4	−1.6	−0.9	−0.3	−0.6	1.8
(P1)CRM-FB	−0.1	−1.0	−1.1	0.1	−0.2	0.1	0.8	0.4	−1.3	−0.3
(P2)CRM-FB	−0.5	−0.1	−0.3	0.3	0.0	0.1	1.1	0.4	1.4	−0.4
(P3)CRM-FB	−0.8	0.7	0.3	−1.4	−1.4	1.1	−1.2	−1.5	0.1	−0.3
(P4)CRM-FB	−0.4	−0.8	−0.4	−0.4	0.3	0.4	0.1	1.1	0.4	−0.7
